# Linking fine-scale behaviour to the hydraulic environment shows behavioural responses in riverine fish

**DOI:** 10.1186/s40462-023-00413-1

**Published:** 2023-08-07

**Authors:** J. Elings, R. Mawer, S. Bruneel, I. S. Pauwels, E. Pickholtz, R. Pickholtz, J. Coeck, M. Schneider, P. Goethals

**Affiliations:** 1 Department of Animal Sciences and Aquatic Ecology, Faculty of Bioscience Engineering, Ghent University, Ghent, Belgium; 2 Research Institute of Nature and Forest (INBO), Brussels, Belgium; 3 sje Ecohydraulic Engineering GmbH, Stuttgart, Germany; 4 Independent Researcher, East Brunswick, NJ USA; 5 School of Zoology, George S. Wise Faculty of Life Sciences, Tel Aviv University, 69978 Tel Aviv, Israel; 6 The Interuniversity Institute for Marine Sciences of Eilat, 88103 Eilat, Israel

**Keywords:** Fish migration, Hidden Markov modelling, Fine-scale acoustic telemetry, Behavioural states, Hydrodynamic modelling

## Abstract

**Background:**

Fish migration has severely been impacted by dam construction. Through the disruption of fish migration routes, freshwater fish communities have seen an incredible decline. Fishways, which have been constructed to mitigate the problem, have been shown to underperform. This is in part due to fish navigation still being largely misunderstood. Recent developments in tracking technology and modelling make it possible today to track (aquatic) animals at very fine spatial (down to one meter) and temporal (down to every second) scales. Hidden Markov models are appropriate models to analyse behavioural states at these fine scales. In this study we link fine-scale tracking data of barbel (*Barbus barbus*) and grayling (*Thymallus thymallus*) to a fine-scale hydrodynamic model. With a HMM we analyse the fish’s behavioural switches to understand their movement and navigation behaviour near a barrier and fishway outflow in the Iller river in Southern Germany.

**Methods:**

Fish were tracked with acoustic telemetry as they approached a hydropower facility and were presented with a fishway. Tracking resulted in fish tracks with variable intervals between subsequent fish positions. This variability stems from both a variable interval between tag emissions and missing detections within a track. After track regularisation hidden Markov models were fitted using different parameters. The tested parameters are step length, straightness index calculated over a 3-min moving window, and straightness index calculated over a 10-min window. The best performing model (based on a selection by AIC) was then expanded by allowing flow velocity and spatial velocity gradient to affect the transition matrix between behavioural states.

**Results:**

In this study it was found that using step length to identify behavioural states with hidden Markov models underperformed when compared to models constructed using straightness index. Of the two different straightness indices assessed, the index calculated over a 10-min moving window performed better. Linking behavioural states to the ecohydraulic environment showed an effect of the spatial velocity gradient on behavioural switches. On the contrary, flow velocity did not show an effect on the behavioural transition matrix.

**Conclusions:**

We found that behavioural switches were affected by the spatial velocity gradient caused by the attraction flow coming from the fishway. Insight into fish navigation and fish reactions to the ecohydraulic environment can aid in the construction of fishways and improve overall fishway efficiencies, thereby helping to mitigate the effects migration barriers have on the aquatic ecosystem.

## Introduction

Migration is an integral part of life for many fish species [1]. Whether for reaching feeding grounds, spawning habitat, or refuge areas, accessibility of different habitats can be crucial for the survival and prosperity of fish communities. As human populations began to develop near riverine ecosystems, so did the impact of human societies on aquatic communities. Human societies began engineering riverine systems as early as 2000BC [2, 3]. Initially, early engineering projects focused on flood protection and irrigation, but as time progressed dam and weir construction for flood control, water storage, and hydropower became more important [3]. Today, rivers in Europe have a barrier every 1.3 km [4]. Fragmentation of water bodies and lack of habitat connectivity is one of the reasons behind an 83% decline in freshwater fish communities and a 76% decline in migratory fish communities since the 1970s [5].

Fishways are often constructed to mitigate migration barriers. Historically, fishway construction has focused on salmon and shad [6] but in recent years a more community-wide approach has begun to emerge [7, 8]. Unfortunately, fishways often do not reach the required efficiencies to support sustainable populations [9–11]. One way to improved total fishway efficiency is by improving the fishway attraction, which measures the proportion of fish successfully finding a fishway. Fishway attraction efficiencies are variable and can range from 36 to 60% for different fishways [9]. One issue faced when aiming to improve fishway attraction can be a lack of understanding regarding the navigational cues used by fish to locate passages when confronted with a barrier [12].

Recent developments in telemetry technology allow researchers to track animals on a near-continuous temporal resolution [13, 14]. With this increase in data-collection possibilities, multiple opportunities arise for analysing fish movement and behaviour [15], especially when movement is linked to fine-scale environmental measurements and modelling [13, 16]. The advent of fine-scale tracking data introduces new complexities to datasets and calls for new developments in data analysis tools. Modelling techniques gaining interest are hidden Markov models (HMMs) and state space models (SSMs) [17, 18]. HMMs (a discrete form of SSMs) have been around since the 1990s [19]. One of the first research projects kickstarting the use of SSMs and HMMs was by Morales et al. [20], where they looked at the movements of elk after a translocation. Recent developments in computational power have led to an increased popularity of these models [17].

HMMs can be used to identify hidden states based on observable variables. Traditionally, these observed variables denote a movement speed (usually expressed in step length) and a straightness parameter (usually expressed in turning angle). However, one is not limited to these parameters and in the field of movement ecology a wide array of movement parameters has been developed [21]. In addition to speed, turning, and their derivatives, other variables, such as dive depth or dive length, can be collected and used in the development of HMMs [22].

A drawback of HMMs is that measurement errors should be negligible. Although the spatio-temporal resolution of aquatic telemetry has become finer, positioning errors due to reflections, receiver time drift, etc. can still be of a magnitude comparable to the distance between neighbour track points. Positioning of acoustic telemetry data can be imprecise and often requires serious pre-processing to result in usable tracks [14, 23]. In particular, for stationary behavioural states (e.g. resting) measurement errors can be problematic as in fine-scale telemetry such states assume a star-shaped movement pattern as result of measurement inaccuracies [14]. Using step length as a proxy for movement speed in such cases can create issues, since the error added to the step lengths will blur the differences between step lengths in resting (generally small steps) versus movement (generally longer steps) states. In turn, this blurring can lead to erroneous state assignments, e.g. by assuming resting behaviour is actually movement due to longer step lengths as a result from measurement errors.

Fish navigation in downstream movement has already been linked to flow direction [24], flow velocity [25, 26] and spatial velocity gradient (SVG) [27]. To our knowledge similar work is lacking for upstream fish navigation. Despite this research gap, it is generally assumed that flow velocity and velocity gradients are equally important in upstream migration as they are in downstream migration [7]. In this research we aim to identify searching behaviour as fish undertake their spawning migration. We focus on barbel (*Barbus barbus*) and grayling (*Thymallus thymallus*) as these species undertake spawning migration and are often confronted with migration barriers [28, 29]. These two fish species were also chosen due to both their abundance within the study system, and their importance in identifying longitudinal river zones [30]. As these fish are presented with a migration barrier, they need to find a fishway to continue their journey and need to navigate using ecohydraulic navigation cues. By linking behavioural switches to these hydraulic patterns, we try to identify which hydraulic cues influence fish navigating upstream to the fishway entrance.

## Methods

### Study site

**Fig. 1:**
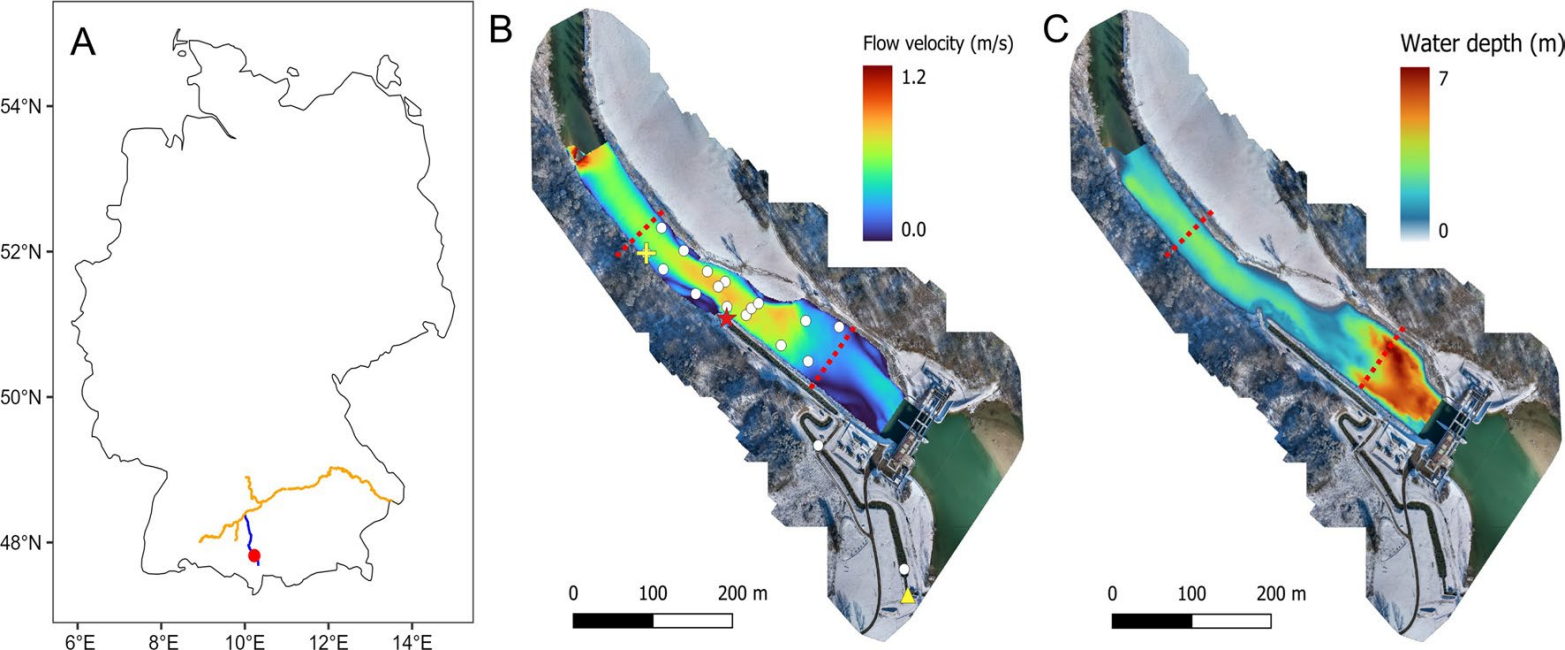
**A** location of the study site (red dot) in Germany. The Iller river (blue) is a right tributary of the Danube (orange). **B** Aerial map of the study site in Altusried overlayed with the simulated flow velocity at a discharge of 50 m^3^/s. White dots show HR2 receiver locations, red star marks the fishway entrance downstream of the hydropower plant, the yellow triangle marks where fish were caught, and the yellow plus-sign marks the release location. **C** Shows the bathymetry of the study site at a discharge of 50 m^3^/s. Water flows from south-east to north-west. Red dotted lines mark the borders of the area for which tag detections were analysed

The study was carried out in the Iller river, a tributary of the Danube, near the town of Altusried, Germany (Fig. 1A). The total discharge during the study period (April-June 2018) was on average 65 m^3^/s and ranged from 27 to 214 m^3^/s. A nature-like fishpass with a discharge capacity of 1 m^3^/s was constructed to mitigate migration over the barrier. The downstream entrance of the fishpass is situated 250 m below the hydropower plant (HPP) on the same side as the turbine outlet. The upstream fishpass entrance is situated 150 m upstream of the HPP (Fig. 1B).

### Hydrodynamic model

**Fig. 2:**
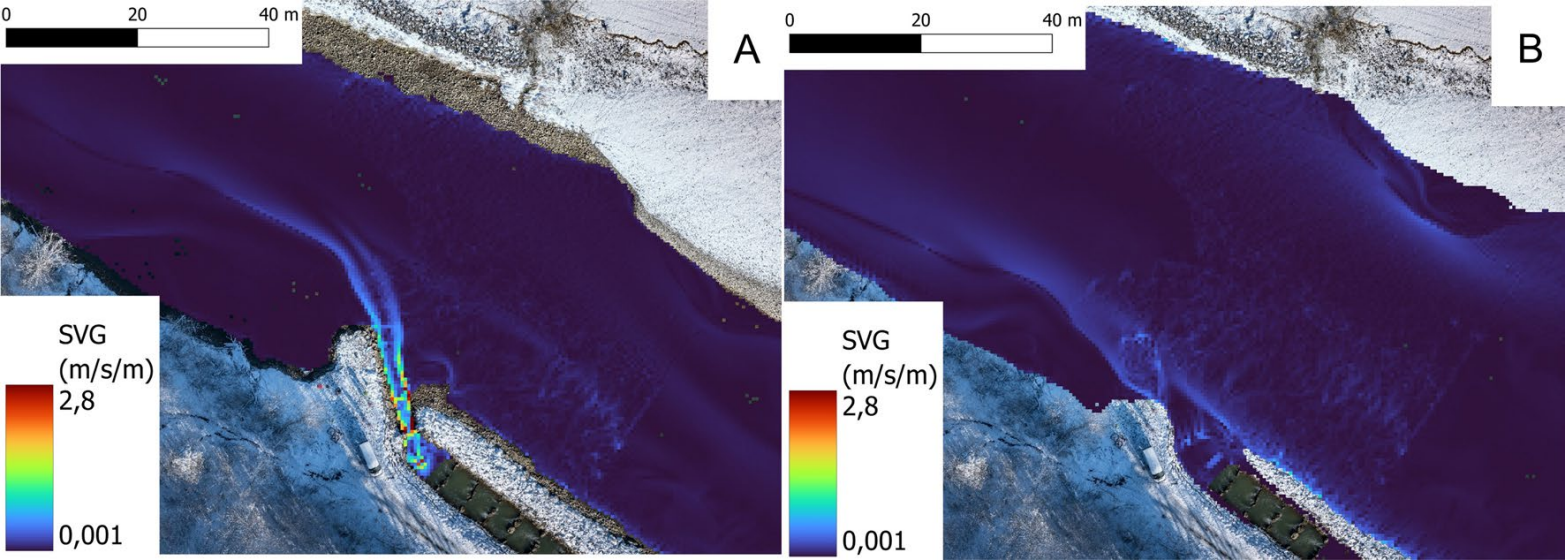
Distribution of SVG at 10 m^3^/s (**A**) and 80 m^3^/s (**B**). At lower discharges the attraction flow of the fishway has a more pronounced effect on SVG than under high discharge conditions

A two-dimensional (2D) hydrodynamic model was developed for eight different discharge situations ranging from 10 to 80 m^3^/s. The modelled values span the range from the minimal discharge needed to operate the HPP, to twice the mean annual flow. Discharge regimes only peaked above these values 3 times within the study period. The model bathymetry is based on echosounder measurements and an aerial drone survey. The computational mesh, consisting of triangle elements, ranging in linear size from 0.25 to 0.5 m, was developed with the pre- and post-processing software SMS [31]. The model Hydro-As_2D [32], based on the Saint-Venant equations, calculates water depths and depth-averaged components of flow velocity for every node of the computation model. Model calibration was done by using water surface elevations at a discharge of 10 m^3^/s; of which 9 m^3^/s passed through the hydropower turbines and 1 m^3^/s through the fishway. In other discharge regimes the fishway remained operating at ~ 1 m^3^/s with the remaining discharge originating from the HPP turbine and/or associated spill weirs. To derive values of SVG on a regular grid, distributions of flow velocity vectors at every discharge were interpolated into a raster nodes on a cell size of 0.5 × 0.5 m (Fig. 2). The SVG is calculated as follows:

$${SVG}_{x}=\frac{\delta vx}{\delta L}$$

$${SVG}_{y}= \frac{\delta vy}{\delta L}$$

$$SVG= \sqrt{{SVG}_{x}^{2}+ {SVG}_{y}^{2}}$$

In which SVG_x_ and SVG_y_ are the SVG components in x and y direction respectively. δvx and δvy are the differences in the flow velocity components between neighbour cells in x and y orthogonal direction respectively, δL is the distance between mesh nodes.

### Fish tagging and tracking

Twenty-two barbel (TL: 498 ± 73 mm; weight: 1356 ± 592 g) and twenty-five grayling (TL: 367 ± 56 mm; 630 ± 270 g) were caught and tagged between March 28th and May 29th 2018 (Table 5). 30 fish were caught in a counting pool within the fishway (47.82°N, 10.23°E; see Fig. 1B), the remaining fish, all grayling, were caught with electrofishing from a boat downstream of the HPP. Fish tagging was done with VEMCO V9-tags (now InnovaSea; random burst interval PPM: 50–70 s; random burst interval HR: 1.1–1.3 s). Tags were implanted in the abdominal cavity after sedation in a 0.2 ml/l 2-phenoxy-ethanol solution. Fish were held in a recovery tank until normal behaviour was shown, typically between 2 and 11 min (see Table 5 in the appendix). After recovery in a holding tank fish were released just downstream of the 2D telemetry array (47.82°N, 10.22°E; Fig. 1B).

The 2D telemetry array consisted of 16 180 kHz HR2 VEMCO receivers and included 6 reference tags. The array spanned 300 m of the river downstream of the hydropower facility (Fig. 1B). Three additional receivers were placed in a 1D set up outside the 2D receiver array to evaluate the behaviour of fish exiting the study area. One receiver was placed 1500 m downstream of the 2D array to evaluate escapement to the downstream section of the river, and two receivers were placed in the fish pass to evaluate fish passage efficiency (one halfway into the fish pass, and one at the fish pass exit upstream of the hydropower facility; see Fig. 1B for receiver placement). The telemetry array was installed from March 2018 to August 2018, but since this study was focussed on migratory behaviour the tracking data was filtered to reflect the migration period of these fish species (see the section ‘Track filtering and regularisation’).

## Data processing and analysis

### Fish positioning

Fish positions were calculated using a novel localization algorithm [33] using time of arrival (TOA) of the tag signals at georeferenced acoustic receivers deployed in the study area (see Fig. 1B). Our method is based on a maximum likelihood formulation as described in [34], where a cost function is defined in terms of time of arrival and sound of speed. Using the recorded time of arrival at each receiver, and given the known distances between receivers and synchronization tags, receivers were synchronized by a polynomial fit for receivers and sound of speed that minimized the sum of absolute residuals across all receiver detections. Contrary to other positioning algorithms, such as YAPS [23], UMAP [35], or VPS [36], this method does not depend on tag transmission interval (e.g., fixed, nominal).

**Fig. 3:**
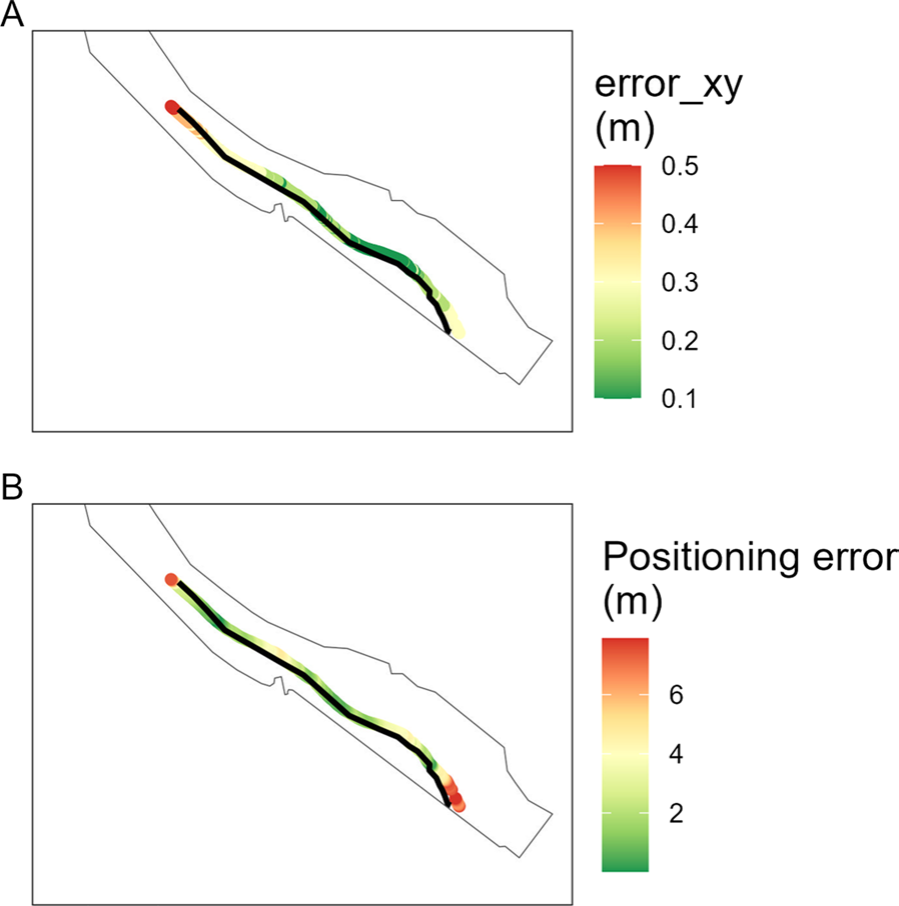
GPS track testing the positioning performance. Error_xy shows higher values towards the edged of the array (**A**). This corresponds to the actual positioning error (**B**)

Fish locations were estimated in a similar manner to the synchronization process above, with the addition of smooth splines components to the horizontal movement of each fish as done by [37]. Based on model performance, estimated errors, in meters, were calculated for each position in the x- and y-direction as well as the overall Cartesian direction (Fig. 3). Based on a GPS test track, the actual mispositioning (*i.e.*, difference between GPS coordinates and the positioning by the algorithm) was highest towards the edges of the array. This corresponded to the error of the overall Cartesian direction values given by the positioning algorithm (see Fig. 3).

### Track filtering and regularisation

To focus on migratory behaviour tracking, data was limited to the spawning period for the two fish species considered in this study. For barbel, tracks between April 20th and June 20th [28] were selected. For grayling, tracks were selected between March 10th to April 20th [29].

Fish positions were retained for the area marked in Fig. 1B. This is the area between the most upstream and downstream receiver in the 2D receiver array. Fish positions outside of this area were removed because the accuracy of the positioning decreases outside of a receiver array [36]. Additionally, positions with an overall calculated Cartesian positioning error (error_xy) exceeding 1.7 m (top 5%) were omitted, to reduce the effect of measurement errors on the subsequent analyses and calculations. Tracks were subdivided and handled as individual tracks if the time-interval between detections exceeded 5 min. Resulting tracks were regularised to a 30 s interval following Lamonica et al. [14]. This was done by fitting a continuous-time correlated random walk to the tracking data and resampling this continuous track with a 30 s interval [38]. The regularised tracks were visually inspected to evaluate potential deviations from the original tracks. Tracks with a duration of less than 10 min or a length of less than 100 m (roughly the distance between the fishway and the edge of the array) were not considered.

### State definitions using HMMs

**Fig. 4:**
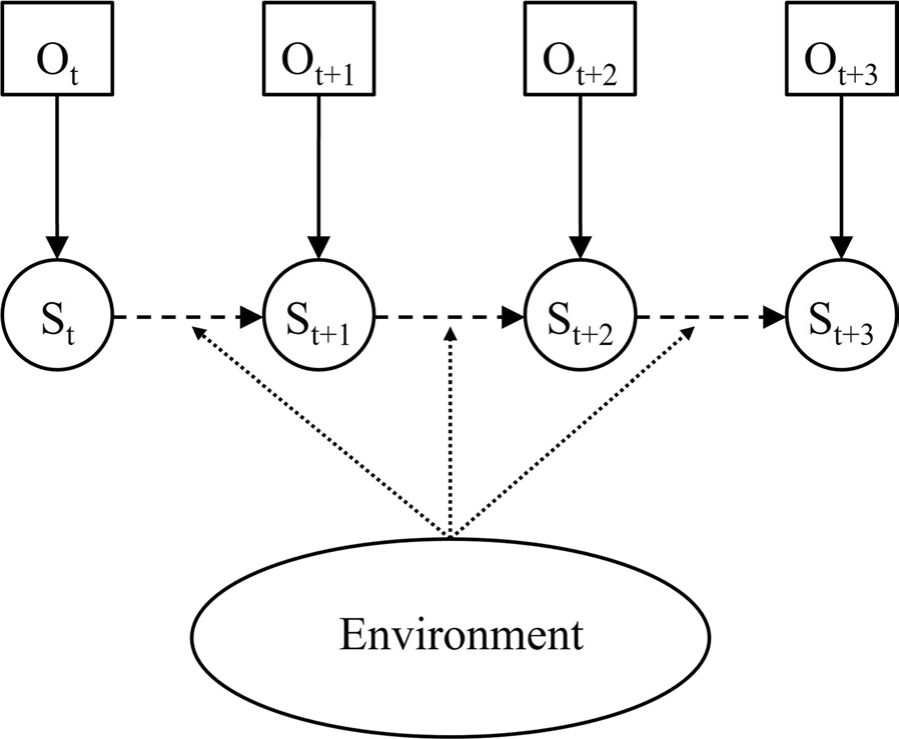
Basic dependence structure of an HMM. The state at time t + 1 (S_t+1_) is dependent on the observation at t + 1 (O_t+1_) through the state distribution, and the state at the previous observation (S_t_) through the transition matrix. The environment can act on the transition matrix and can thus affect the probabilities of a fish changing behaviour between detections. The Viterbi algorithm calculates the most probable state sequence for S_t_:S_t+3_ based on the observation sequence O_t_:O_t+3_ and the transition matrix [39]

HMMs are widely considered to be useful tools in identifying different behavioural patterns from movement data and can help understand the underlying processes [21]. The premise of an HMM is that an unobservable process can be inferred from observable variables (Fig. 4) [ 39]. In the field of movement ecology, this normally means inferring behaviour from movement speed (usually measured in step lengths) and directionality (usually expressed in turning angles). The HMM is dependent on two components: the state distribution and the transition matrix. The state distribution describes the probability of a value being assigned to one state or the other (see Figs. 6 and 9 for examples). The transition matrix shows the probability of a fish changing from one behavioural state to the other. When assigning states to the actual observation, both processes are taken into consideration in the Viterbi algorithm [39, 40], which is used to calculate the most probable state sequence based on the observation sequence.

The straightness index (SI) is a ratio measuring the straightness of a line, where values approaching 0 show complete tortuosity and 1 is perfect straightness. Since it is assumed that resting behaviour is shown as a star-shaped pattern due to telemetry positioning errors, it can be assumed that SI approaches 0 during resting behaviour. Actual movement will be more directed, leading to an SI with higher values. To test the performance of using SI compared to the more commonly used parameter step length, three different 2-state HMMs were developed, one using step lengths as explanatory data stream and two using SI at different time windows (SI_3 with a 3-min window and SI_10 with a 10-min window). In addition to these 2-state models a 3-state model was developed by combining SI_3 and SI_10. The SI was calculated following Batschelet [41] using:

$$SI=\frac{D}{L}$$

In which D stands for the beeline distance between the first and last detection in a moving window, and L for the total track length in said window.

Model parameterization was done with a non-linear minimization (nlm) using the fitHMM function from the momentuHMM package [42]. In the models, step length was assumed to follow a gamma distribution and SI to follow a beta distribution [18, 42]. The beta distribution for SI was chosen as this distribution accurately represents both the 0 to 1 scale, unlike e.g. gamma-distributions, and the continuous nature of the index, unlike e.g. the Bernoulli-distribution. The models were assumed to have two behavioural states and were fitted 50 times with initial values drawn randomly following the methodology of [43]. For each model the iteration with the highest log-likelihood value was retained for further analysis. An inspection of the Viterbi decoded state sequences of the 2-state models indicated that searching behaviour and true resting behaviour could have been merged due to similar SI_10 values. To separate the searching behaviour from the resting behaviour the 2-state SI_10 model was further developed. The choice to improve the SI_10 model was based on AIC values of the different models (see Tables 2 and 4). The state separation of the 3-state model was done by assuming known states for the detections defined as transit behaviour by the 2-state model based on SI_10 and fitting a new model identifying two new states on the remaining, undefined detections based on SI_3.

To test for individual differences an interaction between the transition matrix and fish ID was added In addition to individual differences the effect of catching method was tested for grayling. This was done by fitting separate HMMs were for fish caught in the upstream counting pool and with electrofishing. No differences were found in these model adaptations and thus all fish were pooled by species for the final models.

**Fig. 5:**
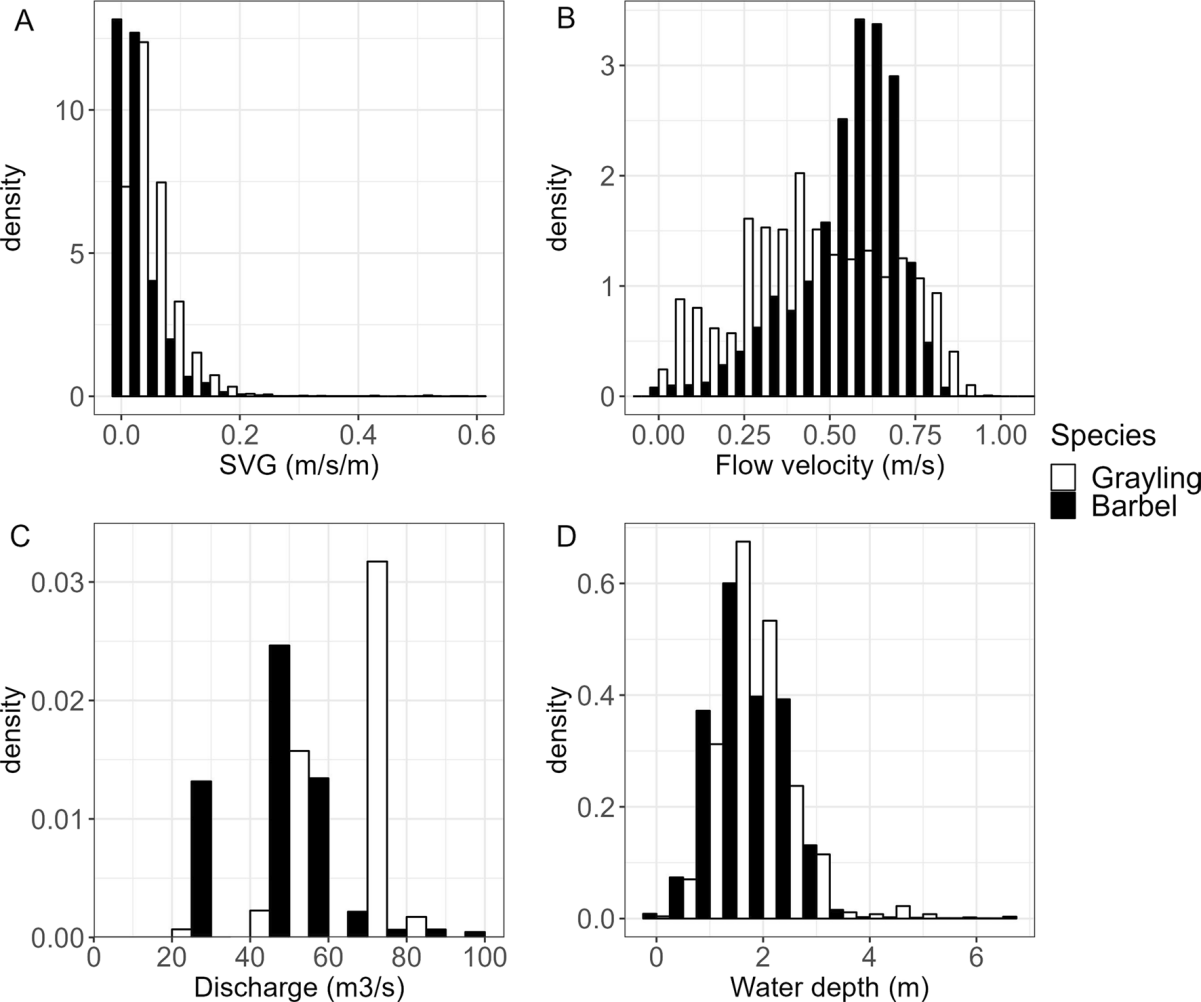
Encountered SVG (**A**), flow velocity (**B**), discharge (**C**), and water depth (**D**) for grayling and barbel

To identify the effect of hydraulic parameters (flow velocity, SVG and their respective angles relative to the fish swimming direction), the best performing model for grayling and the best performing model for barbel were refitted while allowing the hydraulic parameter to act on the transition matrix through a regression formula [42]. Since SVG had a heavily right-skewed distribution (Fig. 5), the effect of higher values was tested. This was done by re-fitting the HMM after removing detections with high SVG values based on the IQR rule. This meant removing detection that exceeded Q_3_ + 3*IQR (the 75^th^ percentile plus 3 times the interquartile range) using the log-transformed data for SVG. As removing these values did not impact the outcome of the HMMs, and SVG values were still within reasonable ranges, it was decided to retain these higher SVG values.

Apart from the hydrodynamic modelling, all data preparation and analysis was done in RStudio v4.2.2 [44].

## Results

### Track information

After pre-processing the fish position data according to the criteria listed in the section ‘Track filtering and regularisation’, 50 tracks remained for grayling and 162 tracks remained for barbel. For grayling the average track duration was 233 min and for barbel average track duration was 152 min. The average track lengths were 1087 m for grayling and 774 m for barbel. See Table 6 in the appendix for detailed information on the tracking data used.

The encountered SVG for both grayling and barbel was heavily right skewed. This can be attributed to the localised nature of high SVG values. High SVG values primarily occurred near the fishway entrance, whereas the rest of the study area was characterised by low values (< 0.1 m/s/m) (Fig. 2).

### Grayling

#### 2-state models

**Fig. 6:**
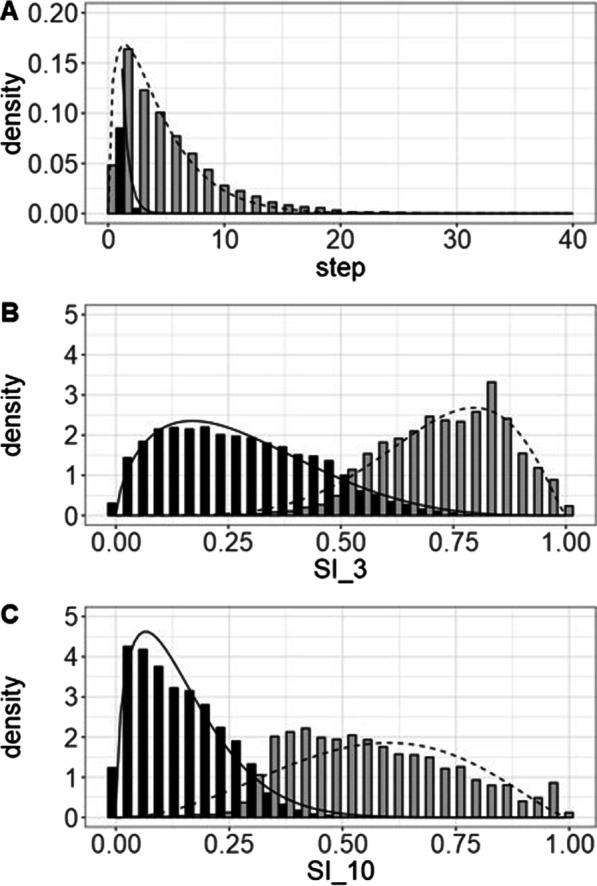
*State* distributions for the 2-state models (**A**) step length model, (**B**) SI_3 model, and (**C**) SI_10 model for grayling. In all models a resting state (solid lines and black bars) and a transit state (dashed lines and grey bars) could be identified

All three 2-state models for grayling showed a resting state and a transit state (see Fig. 6). For the model based on step length, behavioural states were assumed to have a gamma distribution. For the models based on SI, the behavioural states were assumed to follow a beta distribution. In the model based on step lengths the resting state was defined as having a mean step length (µ) of 0.29 m and standard deviation (σ) of 0.37 m. The transit state was defined as a having µ = 4.99 m and σ = 4.29 m. In the SI_3 model the resting state was defined as having µ = 0.28 and σ = 0.02, and the transit state was defined as having µ = 0.72 and σ = 0.01. The SI_10 model had a resting state defined as having µ = 0.15 and σ = 0.01 and a transit state having µ = 0.57 and σ = 0.03 (Fig. 6C).

Based on the AIC values and the log-likelihood, the SI_10 model was the best fit for the data (see Table 2).

#### 3-state model

**Fig. 7:**
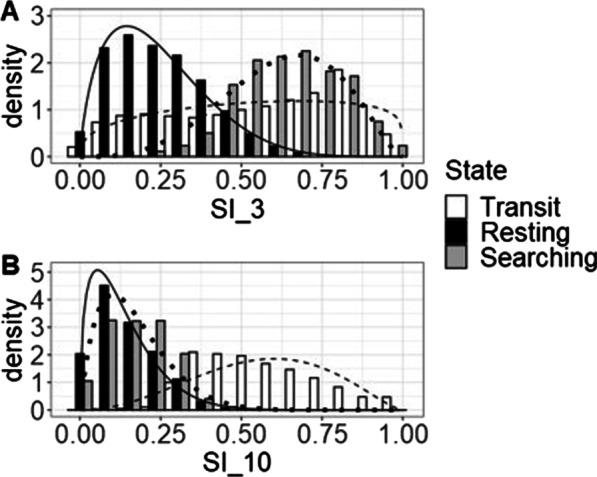
*State* distributions of (**A**) SI_3 and (**B**) SI_10 for the 3-state model for grayling. In the model we see a transit state (white bars and dashed line), a resting state (black bars and solid line), and a searching state (grey bars and dotted line). Even though this model makes biological sense AIC and LL support the 2-state models over this 3-state model

Fixing the transit state from the SI-3 2-state model enabled separating the resting state into a true resting state and a state characterised by searching behaviour (see Fig. 7). The resulting model identified:

1. A straight state, characterized by high SI_3 and high SI_10 values (SI_3: µ = 0.54, σ = 0.27; SI_10: µ = 0.56, σ = 0.18),
2. A very tortuous state, characterized by low SI_3 and SI_10 values (SI_3: µ = 0.24, = 0.14; SI_10: µ = 0.13, σ = 0.09), and
3. A state with medium tortuosity, characterized by high SI_3 but low SI_10 values (SI_3: µ = 0.63 σ = 0.15; SI_10: µ = 0.17, σ = 0.09).

From here on the three states will be referred to as transit, resting, and searching respectively. The 3-state model had a higher AIC and lower LL, and is thus performing poorer, than the two SI models, though it performed better than the step length model (see Table 2).

### Including ecohydraulic variables

**Fig. 8:**
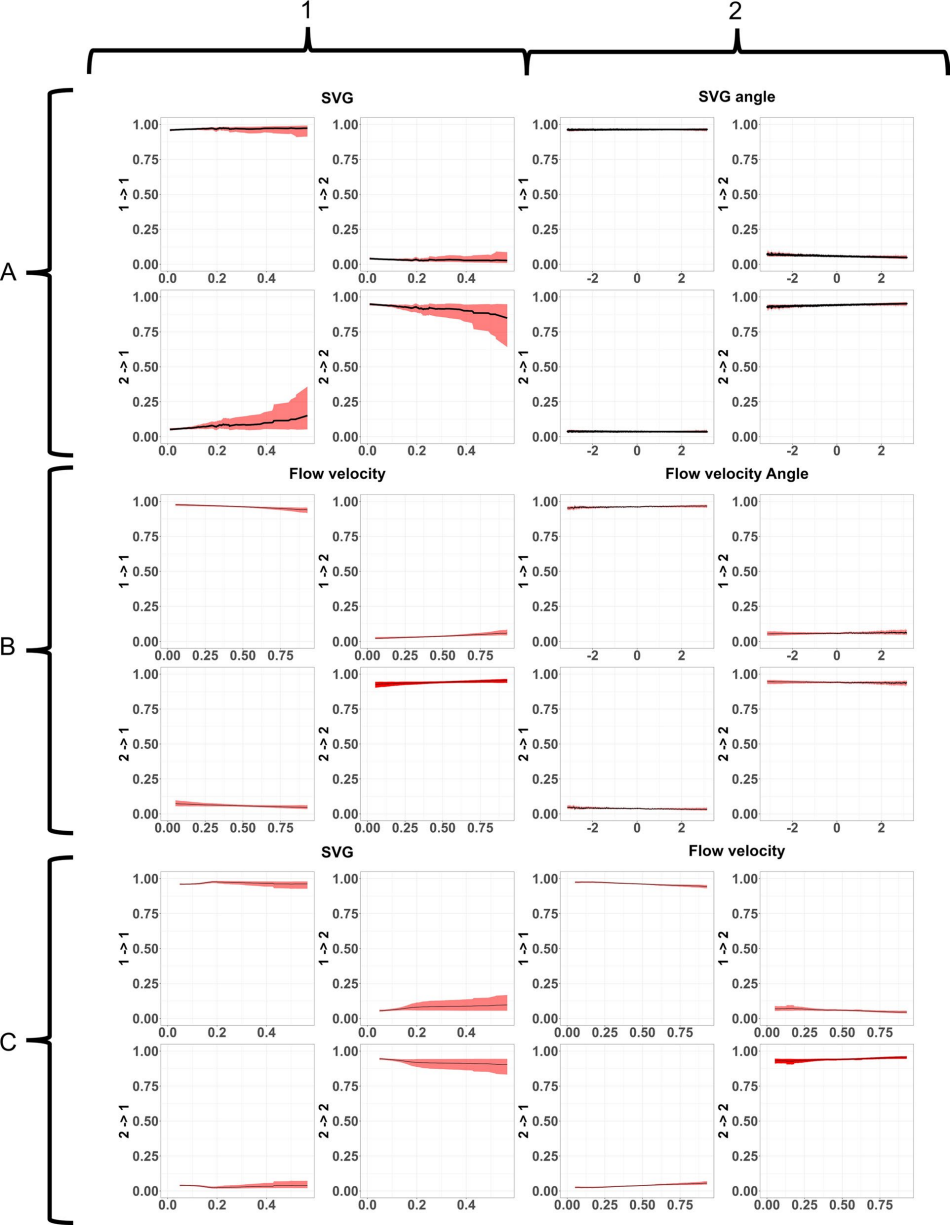
Effect of SVG, flow velocity, and their interaction on the transition matrix for grayling with associated 95% confidence interval (CI) (grey bars). Every cluster of four graphs should be read as: top-left) probability of a behavioural switch from behaviour 1 to behaviour 1, top-right) the chance of switching from behaviour 1 to behaviour 2, bottom-left) switching from behaviour 2 to behaviour 1, and bottom-right) switching from behaviour 2 to behaviour 2. SVG had an effect, though CI is relatively wide (**A1**). SVG relative angle had no effect (**A2**). Flow velocity had no effect for either absolute values (**B1**) or relative angle (**B2**). In the interaction only SVG had an effect (**C1**)

Including SVG affected the transition matrix. As SVG increased, the probability of a fish switching to state 1 (resting) increased. SVG angle along with the interaction between SVG value and SVG angle had a limited effect (Table 1). Including flow velocity and flow direction had a very limited effect (Fig. 8B and Table 1). Including an interaction effect between SVG value and flow velocity results in a slight effect of SVG (Fig. 8C1 and Table 1) whereas flow velocity again had no real effect (Fig. 8C2 and Table 1).

**Table 1:** Regression coefficients for the transition matrix when including SVG, flow, and the interaction between SVG and flow velocity

	1 → 2	2 → 1
*SVG*		
(Intercept)	− 3.20	− 2.94
SVG	− 1.41	2.18
SVG angle	0.09	− 0.04
SVG:SVG angle	− 2.78	− 0.19
*Flow*		
(Intercept)	− 3.47	− 2.62
Flow velocity	0.47	− 0.46
Flow angle	− 0.05	0.13
Flow velocity:Flow angle	− 0.05	− 0.22
*Interaction*		
(Intercept)	− 3.41	− 2.70
SVG	− 1.56	1.21
Flow velocity	0.28	− 0.49
SVG:Flow velocity	5.04	1.67

### Barbel

#### 2-state model

**Fig. 9:**
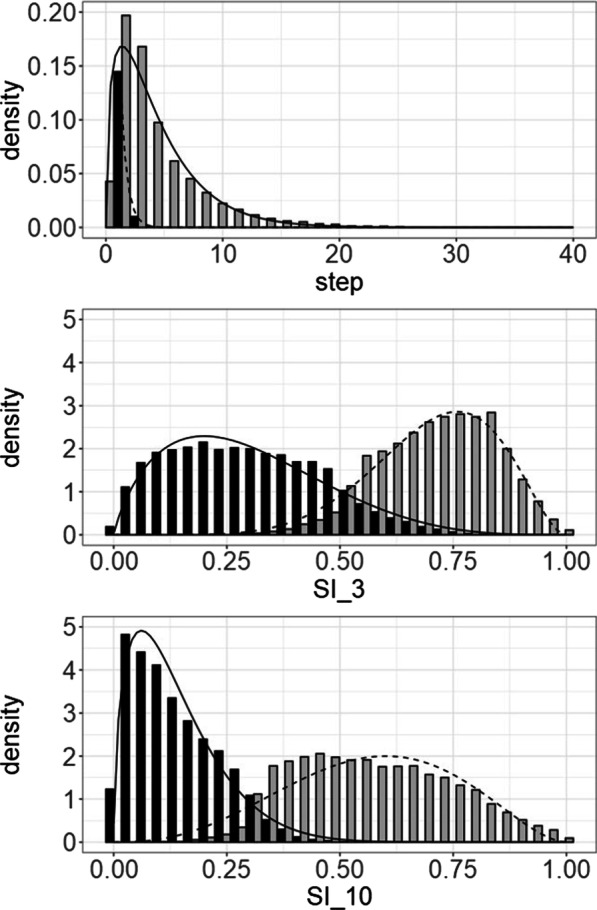
State distributions of the step length model (top), SI_3 model (middle), and the SI_10 model (bottom). All states showed a resting state (black bars and solid lines) and a transit state (grey bars and dashed lines)

For barbel all 2-state models resulted in a resting state and a transit state (see Fig. 9). As with grayling the step length model assumed gamma distributions for the behavioural states and the SI models assumed beta distributions (Tables 2 and 3). In the step length model a resting state found with mean step length of 0.43 m and a standard deviation of 0.51 m and transit behaviour with an average step length of 4.31 m and a standard deviation of 3.55 m. In the SI_3 model the states a tortuous resting state was found with a mean SI of 0.30 m (standard deviation 0.02 m) and a straight transit mode with an average SI of 0.71 m (standard deviation 0.01 m). The SI_10 model also showed a tortuous resting behaviour (µ = 0.14 and σ = 0.01) and a straight transit mode (µ = 0.57 m and σ = 0.02). As with the models developed for grayling the model based on SI_10 performed best with the model based on step length performed poorest (Table 4).

**Table 2:** AIC and log-likelihood (LL) values for the different models for grayling

	AIC	LL
Step length	49781.91	− 24883.96
SI_3	− 11433.97	5723.99
SI_10	− 26147.03	13080.51
3-state	− 7978.57	4011.28
SVG	− 26141.56	13083.78
Flow velocity	− 26150.84	13088.42
Interaction	− 26148.88	13088.42

The 2-state SI_10 model scored best as indicated by AIC and log-likelihood. Including the ecohydraulic variables to the SI_10 model (models SVG, flow velocity, and interaction) did not greatly improve the models as indicated by AIC and log-likelihood values

**Table 3:** Regression coefficients for barbel when including SVG, flow, and the interaction between SVG magnitude and flow velocity

	1 → 2	2 → 1
*SVG*		
(Intercept)	− 3.34	− 3.23
SVG	− 3.16	5.63
SVG angle	0.04	− 0.05
SVG:SVG angle	− 0.24	− 1.40
*Flow*		
(Intercept)	− 3.69	− 2.89
Flow velocity	0.44	− 0.32
Flow angle	− 0.13	0.08
Flow velocity:Flow angle	0.17	− 0.18
*Interaction*		
(Intercept)	− 3.60	− 3.17
SVG	0.71	2.58
Flow velocity	0.46	− 0.17
SVG:Flow velocity	− 7.72	7.14

**Table 4:** AIC and log-likelihood (LL) values for the different models for barbel

	AIC	LL
Step length	142656.72	− 71321.40
SI_3	− 27500.34	13757.17
SI_10	− 60016.88	30015.44
3-state	− 19232.10	9638.05
SVG	− 26155.94	13083.78
Flow velocity	− 26150.84	13088.42
Interaction	− 26148.86	13088.42

The 2-state SI_10 model performed best as indicated by AIC and log-likelihood. Including the ecohydraulic variables to the SI_10 model (models SVG, flow velocity, and interaction) resulted in lower scoring information criteria

#### 3-state model

**Fig. 10:**
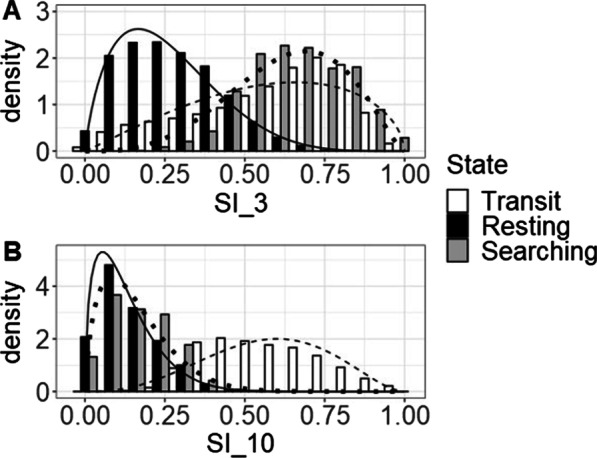
*Beta* distributions of the 3-state model for barbel. The model could identify resting behaviour based on SI_3 and can differentiate between searching and transit based on SI_10

Fitting a 3-state model by fixing the transit-state (state 1 in the 3-state model) resulted in three clear states (see Fig. 10):

1. A straight state, characterized by high SI_3 and high SI_10 values (SI_3: µ = 0. 58, σ = 0. 23; SI_10: µ = 0. 57, σ = 0. 17),
2. A very tortuous state, characterized by low SI_3 and SI_10 values (SI_3: µ = 0. 26, σ = 0.14; SI_10: µ = 0.13, σ = 0.09), and
3. A state with medium tortuosity, characterized by high SI_3 but low SI_10 values (SI_3: µ = 0.65, σ = 0.15; SI_10: µ = 0.16, σ = 0.10).

As with the 3-state model developed for grayling these states will be called transit, resting, and searching respectively. Trying to define a third state decreased the overall performance of the model (Table 4).

#### Including ecohydraulic variables

**Fig. 11:**
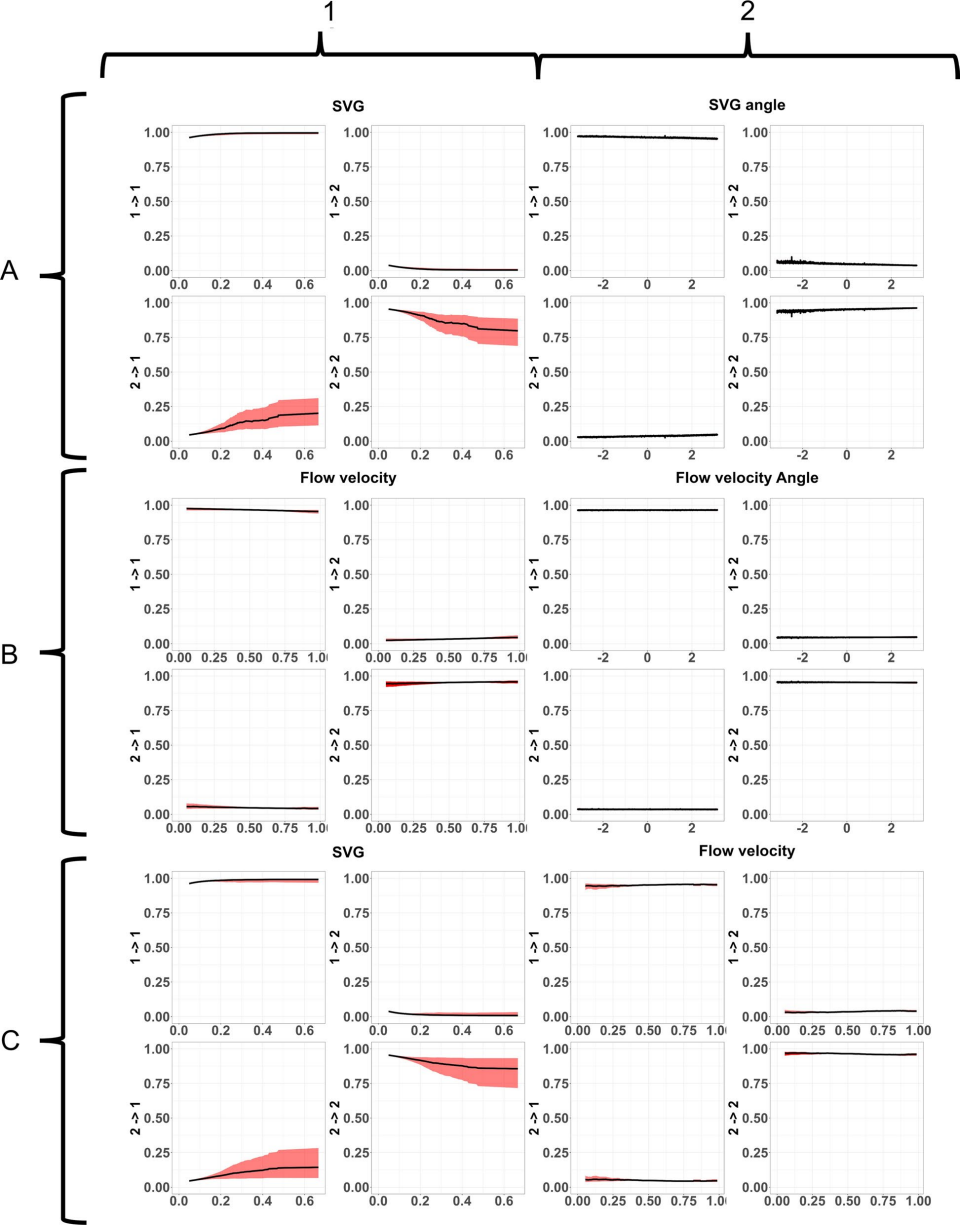
Effect of SVG, flow velocity, and their interaction on the transition matrix for barbel with associated 95% confidence interval (CI) (grey bars). Every cluster of four graphs should be read as: top-left) probability of a behavioural switch from behaviour 1 to behaviour 1, top-right) the chance of switching from behaviour 1 to behaviour 2, bottom-left) switching from behaviour 2 to behaviour 1, and bottom-right) switching from behaviour 2 to behaviour 2. Absolute values of SVG seemed to have an effect, although a wide CI can be seen (**A1**). SVG relative angle had no effect (**A2**). Flow velocity had no effect for either absolute values (**B1**) or relative angle (**B2**). In the interaction only SVG had an effect (**C1**).

The effect of the ecohydraulic parameters were tested on the best performing model, which was the SI_10 model. At high SVG values barbel were more likely to switch behaviour from state 2 (transit) to state 1 (resting). SVG angle (Fig. 11A2), flow velocity (Fig. 11B1), and flow angle (Fig. 11B2) had no effect on the transition probabilities. When testing for the interaction between SVG and flow velocity (Fig. 11C) again only SVG had an effect, but the effect was more pronounced and with a smaller 95% confidence interval, than when testing for SVG and SVG angle.

## Discussion and conclusion

In this paper it is shown that using SI as a data stream to classify behavioural states using HMMs is the better option when compared to using the traditional step length. Based on model AIC and log-likelihood values, the best model for both barbel and grayling was a 2-state model identifying resting and transit behaviour based on a SI calculated over a 10 min window. Trying to identify a third behaviour (searching) diminished model performance.

**Fig. 12:**
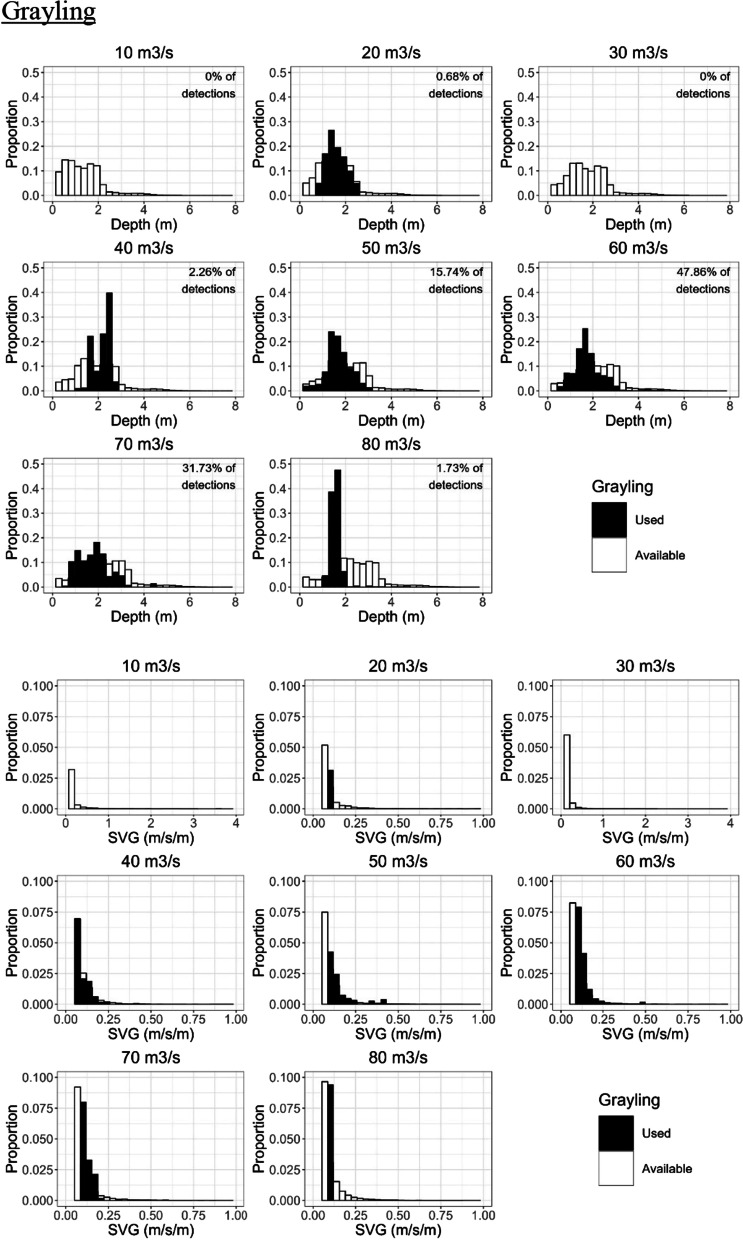

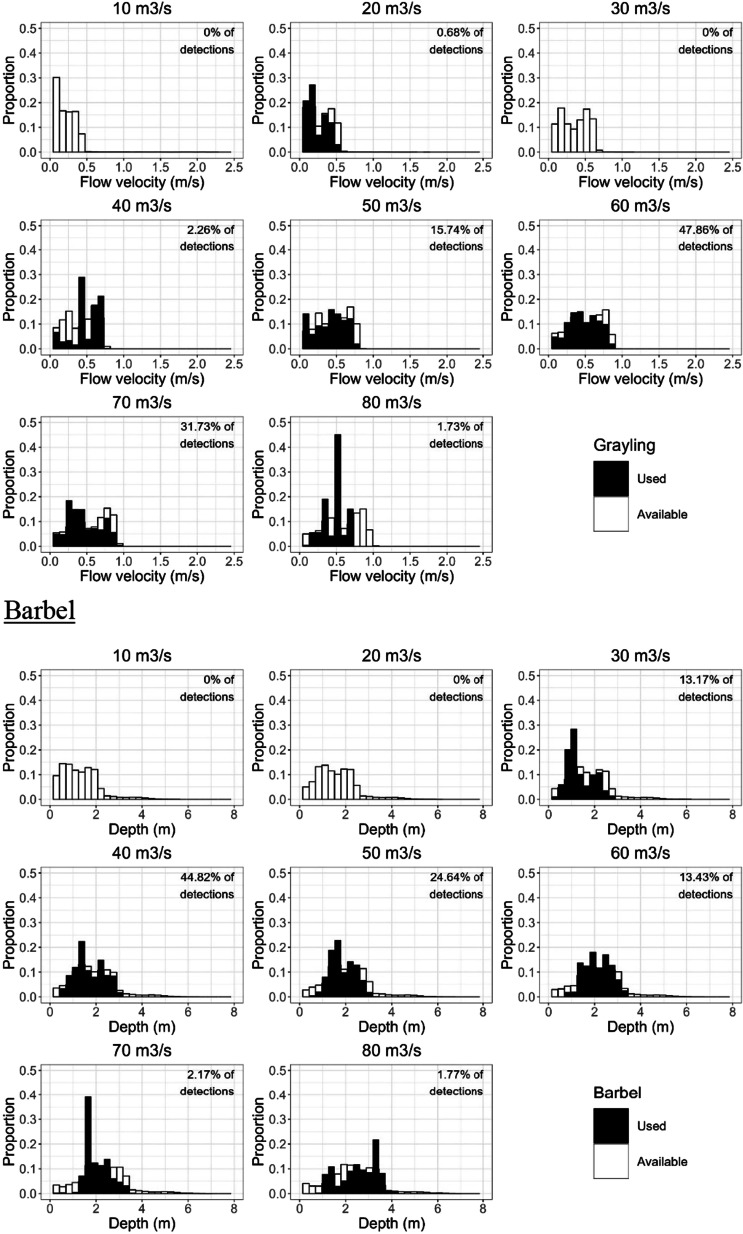

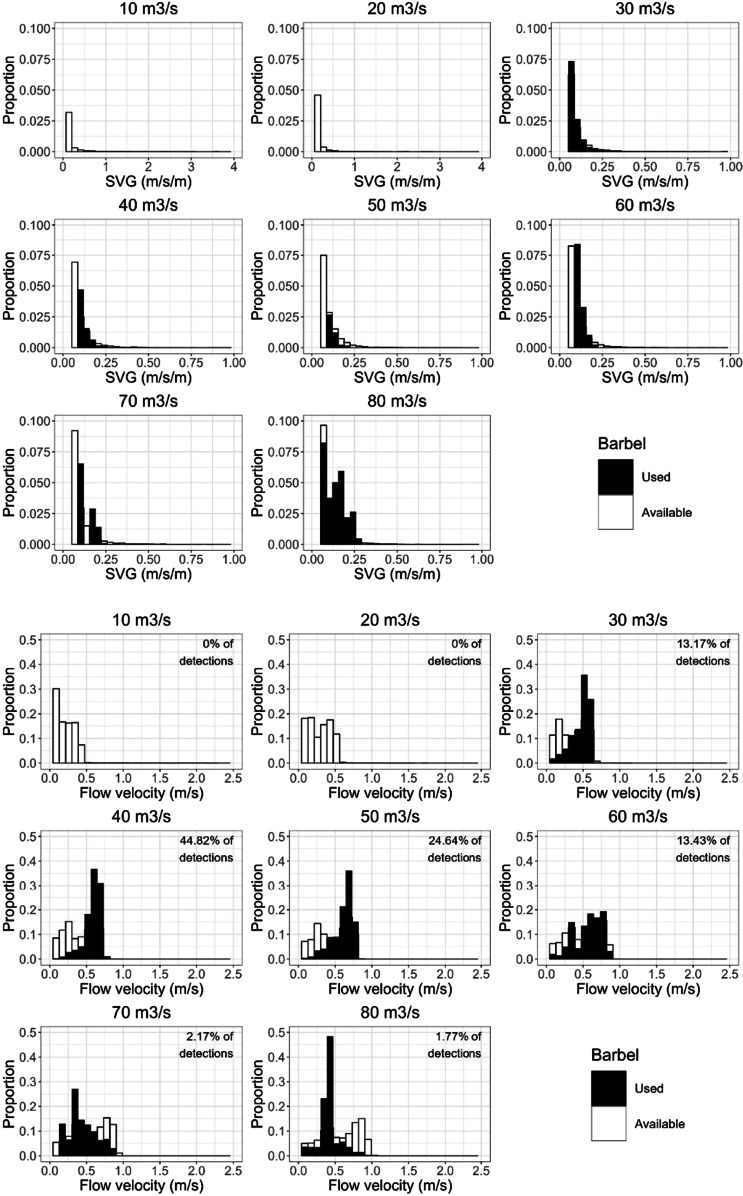
Habitat use (black) and available habitat (white) for grayling and barbel

Linking the behavioural states to ecohydraulic parameters enabled analysing how fish behaviour is affected by the ecohydraulic environment. Surprisingly, fish behaviour was not impacted by flow velocity, contrasting with general assumptions on fish behaviour [7]. The lacking effect of flow velocity might be attributed to the relatively low flow velocities compared to critical swimming speed of the fish species used in this study. Grayling can sustain a swimming speed of 1.3–1.4 m/s for 2 min [45]. For barbel no critical swimming speeds have been found, but the closely related Iberian barbel (*Barbus bocagei*) has a critical swimming speed between 0.7 and 1 m/s [46]. Looking at the used flow velocities it can be seen that barbel indeed tended to select locations with flow velocities of 0.5–0.7 m/s, and only at higher discharges (> 70 m3/s) barbel started selecting lower flow velocities (see Figs. 12 in appendix). Flow velocities ranged from 0.0 to 1.0 m/s, with the highest values around the fishway entrance. Based on critical swimming speeds, grayling should have no problem overcoming the flow velocities in this system. Barbel could potentially have some issues overcoming the flow velocities found in this study site, but from our analyses it seemed that fish in were not affected by the flow velocities found in the Altusried study site. Theoretically, this could be due to barbel being able to utilize habitat structures (for example large boulders) to shelter from high flow velocities or utilize near-bed velocities instead of the full flow velocities seen higher in the water column [47, 48]. However, since this study did not include depth measurements it is impossible to draw definitive conclusions on this subject. Future studies could account for depth by using 3D acoustic tracking when analysing fish navigation in relation to environmental cues such as flow velocity, SVG, bathymetry and substrate.

SVG did have an impact on the behavioural switches. For both grayling and barbel, an increase in SVG was associated with an increased probability of a fish changing behaviour, from transit to resting. However, due to the large window used to calculate SI what we call resting can also include searching behaviour. This is due to the star-shaped patterns approaching similar SI-values as searching behaviour where fish pace up-and-down an area where they expect passage. The effect of SVG on the transition matrices would suggest that SVG is an important navigational cue for fish. However, it should be noted that these effects are accompanied by a wide confidence interval. The wide confidence interval can be attributed to the localized nature of SVG and limited amount of data at higher SVG values. High SVG values almost exclusively occurred near the fishway and are a result of the attraction flow stemming from the fishway. In the tracking data, higher SVG values accounted for very few detections compared to the entire dataset (only 0.15% above 0.3 m/s/m). The effect of these outliers was tested by removing the most extreme cases and re-fitting the model. Removing outliers did not change the broader pattern found when linking SVG to the transition matrix, which in turn led to the decision to retain these values. Since the high SVG values do not represent unrealistic values and their low abundance in the dataset can be attributed by their low abundance in the study system it was also decided to not apply a transformation to handle outliers.

Something that could not be tested sufficiently is the effect of catch location and catching method. Since all barbel were caught in the counting pool in the upper part of the fishway, it can be assumed that these fish already know where the fishway is located. Grayling catches were more evenly distributed between the counting pool and electrofishing. Developing separate HMMs for the two catching methods did not reveal differences in the state definition, leading to all individuals being pooled in the analyses. Studies investigating the learning process of fish when searching for fishways could reveal very interesting findings as laboratory settings have shown spatial learning behaviour in fish [49]. Additionally, individual variation is not directly accounted for in this study. To fully investigate the effect of individual behaviour, a separate HMM would be required for every individual. Since HMMs are a data-driven method, this would rely on all behavioural types being shown by all fish. As not all fish were exhibiting all different behaviours, and similar behaviour might look different for different fish it is difficult to make individual HMMs universal. Including fish ID as a random variable affecting the transition matrix did not reveal individual differences. This indicates that behavioural switches are similar between different fish of the same species.

For fishway design, results suggest that the focus should be on optimizing SVG rather than flow velocity in the attraction flow. However, given that SVG is a direct effect of (differences in) flow velocity, this does not mean that flow velocity can be completely removed from the equation. First and foremost, attraction flow velocity should not exceed swimming capacities of target fish species [50]. In addition, a high SVG value depends on areas with higher flow velocities adjacent to areas with lower flow velocities. So rather than absolute values, flow velocity should be considered in relation to the surrounding ecohydraulic environment and how target species react to gradients in this flow velocity. In this study, the application of HMMs to fine-scale tracking data is demonstrated. Yet, attributes of fine-scale data still need to be considered. Positioning errors can seriously affect model performance when these models are based on parameters derived from only a few positions. For example, step length only depends on two detections and are prone to error at fine scales [51]. Taking parameters calculated over more detections can smooth the effects of positioning errors. Therefore, we recommend that other movement parameters are used when applying HMMs to fine-scale tracking data, preferably parameters that are calculated over multiple detections. Applying such an approach allows researchers to smooth the tracking data while still retaining valuable information on the very fine scale, enabling links to be made between animal movement and the environment the animal utilizes [15]. SI seems to be a very viable option to use in applying these models, but there is a wide variety of parameters (e.g. net squared displacement, sinuosity, multi-scale straightness index) that can be used in defining behavioural states [21]. Important steps to consider when applying these kinds of models is thoroughly understanding your study system, potential causes of mispositioning, and the effect the temporal resampling and extent of the moving window has when calculating the movement parameters used in the model.

## Data Availability

Data and code can be requested with the corresponding author.

## Appendix

See Fig. 12.

See Tables

**Table 5:** Information on the tagged fish in this study

Tagging date	Species	Catch method	Total length (mm)	Weight (g)	Recovery time
29/05/2018	Barbel	Counting pool	570	2160	0:03:49
29/05/2018	Barbel	Counting pool	594	2161.7	0:04:03
29/05/2018	Barbel	Counting pool	456	932.6	0:03:37
24/05/2018	Barbel	Counting pool	504	1285.8	0:03:43
24/05/2018	Barbel	Counting pool	496	1106.8	0:07:34
24/05/2018	Barbel	Counting pool	460	911.2	0:03:37
24/05/2018	Barbel	Counting pool	619	2343.3	0:07:34
24/05/2018	Barbel	Counting pool	545	1567.6	0:06:30
29/05/2018	Barbel	Counting pool	480	1180.5	0:03:58
24/05/2018	Barbel	Counting pool	526	1465.7	0:05:04
24/05/2018	Barbel	Counting pool	330	327.2	0:04:45
24/05/2018	Barbel	E-fishing	460	1053.2	0:05:04
24/05/2018	Barbel	Counting pool	461	1010.6	0:06:19
24/05/2018	Barbel	Counting pool	585	2105.1	0:11:58
24/05/2018	Barbel	Counting pool	594	2212.7	0:03:17
24/05/2018	Barbel	Counting pool	513	1544.1	0:07:15
24/05/2018	Barbel	E-fishing	457	822.9	0:05:26
17/05/2018	Barbel	Counting pool	516	1306.5	0:06:36
17/05/2018	Barbel	Counting pool	387	648.4	0:06:01
17/05/2018	Barbel	Counting pool	545	2074.4	0:05:57
17/05/2018	Barbel	Counting pool	466	1009.6	0:06:12
17/05/2018	Barbel	Counting pool	400	621.2	0:08:47
11/04/2018	Grayling	E-fishing	403	673.3	0:11:23
11/04/2018	Grayling	Counting pool	409	623.2	0:07:13
11/04/2018	Grayling	NA	350	315	0:10:37
11/04/2018	Grayling	Counting pool	326	262.1	0:11:12
11/04/2018	Grayling	Counting pool	439	755.4	0:03:58
04/04/2018	Grayling	E-fishing	420	791.3	0:04:59
11/04/2018	Grayling	Counting pool	508	1250.8	0:04:42
11/04/2018	Grayling	Counting pool	426	756.8	0:08:37
11/04/2018	Grayling	E-fishing	416	843.9	0:06:00
11/04/2018	Grayling	Counting pool	411	640.1	0:02:53
04/04/2018	Grayling	E-fishing	348	402.1	0:05:03
04/04/2018	Grayling	E-fishing	383	657.7	0:04:57
04/04/2018	Grayling	E-fishing	431	712.3	0:05:03
04/04/2018	Grayling	Counting pool	371	444.6	0:05:28
04/04/2018	Grayling	E-fishing	494	1048.5	0:05:51
04/04/2018	Grayling	Counting pool	425	642.4	0:05:54
04/04/2018	Grayling	E-fishing	498	1152.9	0:11:02
04/04/2018	Grayling	Counting pool	331	317.3	0:03:50
04/04/2018	Grayling	E-fishing	400	683	0:03:55
04/04/2018	Grayling	E-fishing	389	634.2	0:03:30
04/04/2018	Grayling	E-fishing	406	707.3	0:05:19
04/04/2018	Grayling	E-fishing	309	233.4	0:04:46
04/04/2018	Grayling	Counting pool	407	606.4	0:02:57
28/03/2018	Grayling	E-fishing	318	312	0:08:21
28/03/2018	Grayling	E-fishing	304	286	0:07:29

^5^ and

**Table 6:** Information on the individual tracks used in the analyses

Species	Track id	Number of detections	Duration (minutes)	Total track length (m)	Maximum displacement (m)
Grayling	46,868–15	511	255	867.69	148.35
Grayling	46,868–16	655	327	2123.69	274.57
Grayling	46,868–19	1940	969.5	3559.65	117.64
Grayling	46,868–5	102	50.5	680.98	104.23
Grayling	46,868–7	170	84.5	247.79	134.33
Grayling	46,869–1	459	229	2790.99	105.76
Grayling	46,901–15	103	51	222.20	151.63
Grayling	46,901–18	70	34.5	400.14	146.14
Grayling	46,901–19	141	70	696.41	114.77
Grayling	46,901–22	348	173.5	859.76	141.54
Grayling	46,901–31	62	30.5	258.77	122.00
Grayling	46,901–34	267	133	1167.22	102.70
Grayling	46,901–38	1425	712	3808.62	126.63
Grayling	46,902–1	76	37.5	651.77	159.04
Grayling	46,903–1	85	42	452.06	221.20
Grayling	46,903–2	189	94	523.37	143.65
Grayling	46,905–10	180	89.5	240.69	100.64
Grayling	46,905–30	259	129	798.70	114.96
Grayling	46,905–47	338	168.5	866.60	157.51
Grayling	46,905–49	323	161	566.98	158.02
Grayling	46,905–55	316	157.5	590.84	133.08
Grayling	46,905–6	1067	533	1100.80	100.51
Grayling	46,906–10	715	357	2501.92	207.39
Grayling	46,906–17	318	158.5	2716.77	232.77
Grayling	46,906–18	319	159	2070.71	134.79
Grayling	46,906–19	613	306	1967.08	268.14
Grayling	46,906–2	243	121	1066.06	127.60
Grayling	46,906–21	1228	613.5	1054.39	156.74
Grayling	46,906–22	92	45.5	799.70	272.43
Grayling	46,906–47	257	128	1287.69	275.00
Grayling	46,906–51	176	87.5	1300.26	139.50
Grayling	46,907–19	86	42.5	525.89	106.73
Grayling	46,908–14	286	142.5	177.55	109.41
Grayling	46,908–37	829	414	1266.41	206.48
Grayling	46,908–90	587	293	1074.50	194.73
Grayling	46,908–94	764	381.5	1070.96	146.99
Grayling	46,908–95	84	41.5	773.02	206.97
Grayling	46,909–9	666	332.5	1125.09	168.90
Grayling	46,910–12	98	48.5	566.11	171.65
Grayling	46,910–27	1346	672.5	1922.38	130.16
Grayling	46,910–40	372	185.5	572.78	152.55
Grayling	46,910–49	330	164.5	1590.07	280.60
Grayling	46,910–55	698	348.5	1039.94	153.00
Grayling	46,910–64	310	154.5	732.75	215.44
Grayling	46,910–66	308	153.5	655.33	274.43
Grayling	46,911–2	67	33	407.23	101.82
Grayling	46,913–5	69	34	339.50	253.44
Grayling	46,914–12	152	75.5	506.37	197.37
Grayling	46,914–5	2804	1401.5	1236.82	128.31
Grayling	46,914–9	417	208	461.67	122.91
Barbel	46,838–11	190	94.5	611.42	250.74
Barbel	46,838–28	127	63	511.66	281.67
Barbel	46,838–29	116	57.5	453.16	228.40
Barbel	46,838–3	350	174.5	868.40	126.88
Barbel	46,838–35	370	184.5	718.88	240.70
Barbel	46,838–36	543	271	830.03	129.74
Barbel	46,838–5	145	72	408.97	167.13
Barbel	46,838–8	295	147	871.92	246.96
Barbel	46,839–10	882	440.5	1083.63	182.55
Barbel	46,839–13	386	192.5	842.06	199.33
Barbel	46,839–15	222	110.5	562.25	146.39
Barbel	46,839–17	100	49.5	317.26	136.76
Barbel	46,839–18	448	223.5	506.04	105.05
Barbel	46,839–22	116	57.5	751.54	252.02
Barbel	46,839–28	102	50.5	282.41	133.86
Barbel	46,839–30	242	120.5	1059.66	235.88
Barbel	46,839–32	387	193	642.94	197.88
Barbel	46,839–35	80	39.5	501.65	201.21
Barbel	46,839–37	354	176.5	440.42	209.18
Barbel	46,840–6	904	451.5	673.52	184.55
Barbel	46,840–7	176	87.5	377.22	210.40
Barbel	46,840–8	383	191	535.65	205.15
Barbel	46,844–11	93	46	482.80	185.23
Barbel	46,844–4	615	307	579.47	125.11
Barbel	46,844–7	316	157.5	553.36	226.63
Barbel	46,845–24	190	94.5	798.79	120.05
Barbel	46,845–27	103	51	468.00	175.34
Barbel	46,845–34	523	261	1246.42	128.36
Barbel	46,845–39	377	188	519.22	132.66
Barbel	46,845–5	305	152	310.52	119.61
Barbel	46,845–6	373	186	462.12	177.54
Barbel	46,845–7	71	35	300.43	262.76
Barbel	46,845–9	175	87	910.72	233.93
Barbel	46,846–2	1398	698.5	2977.61	120.33
Barbel	46,846–20	141	70	508.31	211.11
Barbel	46,846–4	339	169	927.15	159.55
Barbel	46,846–5	80	39.5	468.20	249.80
Barbel	46,846–8	364	181.5	475.79	118.89
Barbel	46,847–101	547	273	1773.41	115.75
Barbel	46,847–102	563	281	1372.83	116.01
Barbel	46,847–104	87	43	370.50	270.41
Barbel	46,847–105	776	387.5	1082.35	113.16
Barbel	46,847–106	163	81	392.77	191.74
Barbel	46,847–131	66	32.5	334.98	263.56
Barbel	46,847–137	192	95.5	427.44	125.43
Barbel	46,847–138	699	349	1016.05	265.88
Barbel	46,847–142	74	36.5	297.46	252.19
Barbel	46,847–2	733	366	468.78	223.34
Barbel	46,847–26	108	53.5	431.19	158.95
Barbel	46,847–29	75	37	267.25	135.84
Barbel	46,847–4	165	82	207.95	122.47
Barbel	46,847–57	193	96	899.50	276.68
Barbel	46,847–58	70	34.5	290.39	227.57
Barbel	46,847–77	196	97.5	243.28	106.58
Barbel	46,848–10	184	91.5	1173.15	184.49
Barbel	46,848–11	325	162	1681.92	277.47
Barbel	46,848–12	101	50	587.19	165.22
Barbel	46,848–15	108	53.5	561.42	208.67
Barbel	46,848–2	228	113.5	175.27	107.89
Barbel	46,848–3	143	71	238.16	130.36
Barbel	46,849–10	280	139.5	662.69	145.93
Barbel	46,849–11	84	41.5	654.58	227.49
Barbel	46,849–12	83	41	315.35	274.92
Barbel	46,849–19	819	409	613.40	125.00
Barbel	46,849–20	611	305	536.25	138.41
Barbel	46,849–21	518	258.5	745.78	215.62
Barbel	46,849–24	199	99	265.20	122.80
Barbel	46,849–25	483	241	1167.38	147.45
Barbel	46,849–3	328	163.5	1374.70	150.04
Barbel	46,849–4	119	59	174.61	124.38
Barbel	46,849–7	118	58.5	576.44	130.93
Barbel	46,849–9	531	265	1046.08	241.60
Barbel	46,850–11	416	207.5	640.76	270.75
Barbel	46,850–13	65	32	317.14	117.33
Barbel	46,850–6	166	82.5	600.99	249.76
Barbel	46,851–27	63	31	279.17	172.87
Barbel	46,851–39	100	49.5	280.03	122.18
Barbel	46,851–41	73	36	392.06	214.83
Barbel	46,851–43	128	63.5	438.04	257.09
Barbel	46,851–45	73	36	384.18	251.18
Barbel	46,851–5	84	41.5	383.96	257.24
Barbel	46,851–53	80	39.5	338.02	255.87
Barbel	46,852–2	659	329	639.43	129.91
Barbel	46,852–3	1664	831.5	2100.91	141.61
Barbel	46,852–4	340	169.5	364.24	134.73
Barbel	46,852–7	199	99	1334.10	277.14
Barbel	46,852–8	985	492	2374.94	208.67
Barbel	46,852–9	161	80	858.97	277.20
Barbel	46,853–11	154	76.5	658.67	251.51
Barbel	46,853–14	109	54	743.68	238.13
Barbel	46,853–15	164	81.5	611.83	186.28
Barbel	46,853–2	89	44	419.99	119.09
Barbel	46,853–21	122	60.5	547.81	141.41
Barbel	46,853–22	632	315.5	745.14	182.10
Barbel	46,853–8	210	104.5	1516.06	270.84
Barbel	46,853–9	101	50	674.40	123.75
Barbel	46,854–114	401	200	584.52	237.75
Barbel	46,854–123	231	115	484.11	177.77
Barbel	46,854–124	209	104	390.18	127.34
Barbel	46,854–131	74	36.5	430.19	246.19
Barbel	46,854–135	366	182.5	940.50	191.35
Barbel	46,854–14	72	35.5	438.03	272.69
Barbel	46,854–15	83	41	724.78	284.07
Barbel	46,854–21	100	49.5	710.50	210.45
Barbel	46,854–23	71	35	387.82	120.51
Barbel	46,854–27	94	46.5	475.64	246.98
Barbel	46,854–30	68	33.5	485.96	155.25
Barbel	46,854–4	91	45	346.96	127.89
Barbel	46,854–41	156	77.5	1202.55	280.31
Barbel	46,854–44	85	42	487.17	148.41
Barbel	46,854–46	204	101.5	1402.73	262.26
Barbel	46,854–47	168	83.5	707.82	208.23
Barbel	46,854–48	218	108.5	1221.04	150.20
Barbel	46,854–49	522	260.5	820.55	200.81
Barbel	46,854–50	71	35	299.12	248.36
Barbel	46,854–51	732	365.5	1166.61	247.99
Barbel	46,854–54	1342	670.5	2958.65	228.94
Barbel	46,854–57	217	108	427.84	245.54
Barbel	46,854–59	103	51	500.45	251.33
Barbel	46,854–79	63	31	318.54	100.96
Barbel	46,854–84	133	66	597.13	244.27
Barbel	46,855–14	220	109.5	608.47	103.13
Barbel	46,855–15	101	50	405.30	139.32
Barbel	46,855–16	157	78	1321.74	203.87
Barbel	46,855–21	102	50.5	600.15	292.26
Barbel	46,856–3	195	97	1084.99	217.91
Barbel	46,856–4	95	47	424.59	174.03
Barbel	46,856–5	84	41.5	495.96	215.96
Barbel	46,857–36	804	401.5	1107.25	178.93
Barbel	46,858–102	95	47	523.25	256.18
Barbel	46,858–104	96	47.5	202.38	135.76
Barbel	46,858–105	357	178	1159.60	131.84
Barbel	46,858–108	211	105	601.96	207.37
Barbel	46,858–117	902	450.5	3482.30	256.81
Barbel	46,858–119	1016	507.5	1698.74	222.88
Barbel	46,858–124	181	90	607.52	230.18
Barbel	46,858–129	117	58	644.67	270.40
Barbel	46,858–131	206	102.5	468.96	183.87
Barbel	46,858–2	651	325	1022.17	268.67
Barbel	46,858–24	85	42	400.54	136.77
Barbel	46,858–30	72	35.5	483.89	200.37
Barbel	46,858–46	324	161.5	1154.56	227.68
Barbel	46,858–48	378	188.5	712.82	237.56
Barbel	46,858–5	238	118.5	596.84	200.52
Barbel	46,858–59	109	54	699.26	247.25
Barbel	46,858–6	249	124	593.89	277.85
Barbel	46,858–60	126	62.5	596.29	268.63
Barbel	46,858–63	224	111.5	1078.85	225.83
Barbel	46,858–69	68	33.5	520.35	164.40
Barbel	46,858–71	203	101	1310.52	252.91
Barbel	46,858–72	71	35	273.86	128.92
Barbel	46,858–80	424	211.5	2095.30	186.68
Barbel	46,858–81	82	40.5	527.62	112.18
Barbel	46,858–82	111	55	497.77	135.57
Barbel	46,858–84	1667	833	4021.09	226.92
Barbel	46,858–85	624	311.5	1760.76	253.25
Barbel	46,858–92	171	85	806.29	265.10
Barbel	46,858–95	149	74	565.15	251.89
Barbel	46,859–2	1024	511.5	1553.12	210.82
Barbel	46,859–8	1882	940.5	3139.94	109.30
Barbel	46,859–9	244	121.5	622.37	153.20
Barbel	46,860–3	223	111	578.69	142.95

^6^.
